# Improving the measurement of adult mortality

**DOI:** 10.2471/BLT.24.293074

**Published:** 2025-06-10

**Authors:** Ashira Menashe-Oren, Martin Nyahoda, Romesh Silva, Martin Bratschi, Ruxana Jina, Philip W Setel, Adam Karpati, Bruno Masquelier, Petra Nahmias, Afsaneh Yazdani, Tanja Sejersen, Haoyi Chen, Stephane Helleringer

**Affiliations:** a Centre for Demographic Research, Catholic University of Louvain, Place Montesquieu 1, Louvain-la-Neuve, 1348 Belgium.; b School of Veterinary Medicine, University of Zambia, Lusaka, Zambia.; c Population and Development Branch, United Nations Population Fund, New York, United States of America (USA).; d Vital Strategies, New York, USA.; e Statistics Division, United Nations Economic and Social Commission for Asia and the Pacific, Bangkok, Thailand.; f Statistics Division, United Nations Department of Economic and Social Affairs, New York, USA.; g Division of Social Science, New York University Abu Dhabi, Abu Dhabi, United Arab Emirates.

The Measuring Adult Mortality Community of Practice is a new global network whose aim is to improve our knowledge of adult mortality, that is, the risk of dying at age 15 years and older. The network is open to a variety of individuals engaged in the production of mortality data, in national statistical offices, health ministries and local government, research institutions and/or civil society organizations; it currently has over 140 members. The network was established in 2023 with initial funding from the United Nations Economic and Social Commission for Asia and the Pacific, and the Data for Health Initiative of Bloomberg Philanthropies.

The network emerged to address the major challenges in producing accurate and timely statistics on adult mortality – challenges that are particularly concentrated in low- and middle-income countries, where data remain scarce. The coronavirus disease 2019 (COVID-19) pandemic, a health crisis that disproportionately affected older adults, highlighted the issue of data scarcity, as many countries were unable to track the full range of excess deaths in their populations.^1^ Data scarcity in low- and middle-income countries is expected to be exacerbated by the recent decision by the United States government to end support for the Demographic Health Surveys (DHS), risking the loss of one of the main sources of information on adult deaths in African and Asian countries. Furthermore, gaps in adult mortality data are problematic for tracking progress towards the sustainable development goals (SDG) because several indicators emphasize causes of deaths that primarily affect adults. These indicators include noncommunicable diseases (target 3.4), acquired immunodeficiency syndrome (target 3.3), suicides (target 3.4) or road traffic accidents (target 3.6), as well as exposures that have an impact on survival throughout the life course, such as air pollution and access to water (targets 3.9 and 6.3). Beyond 2030, adult deaths will become an even more salient component of overall mortality, with the proportion of deaths in low- and middle-income countries occurring for populations older than 15 years projected to reach 89.7% in 2030 and 94.5% in 2050 – up from 70.6% in 2000.^2^

Ideally, adult mortality is measured by combining complete counts of deaths continuously generated by civil registration and vital statistics systems and full enumerations of the population at risk of death obtained from recent censuses (Table 1). However, civil registration and vital statistics systems remain largely deficient in many low- and middle-income countries – they only register a fraction of all deaths.^3^ Low registration is due to multiple barriers including insufficient civil infrastructure, limited awareness of registration requirements among local populations, high transportation, vague potential benefits associated with registration and limited incentives to complete administrative procedures. When deaths are registered, their cause(s) might frequently remain unknown or inaccurate, particularly when the death was not attended by a physician, or because physicians and other staff lack the training and/or equipment required to investigate and document what people died from.

**Table 1:** Methods for measuring adult mortality in low- and middle-income countries

Method and scope	Approach	Periodicity	Indicator	Weakness	Extension and/or innovation
**Civil registration**					
All deaths in defined administrative areas (such as country, regions, districts)	Registration by qualified informants at local registration offices; and/or Notifications from health facilities and other agencies (such as police)	Continuous	Crude death rate; Counts of deaths by age, sex and cause; and Age-sex-and-cause specific rates, when combined with census and/or register data	Low or very low death registration rates in many low- and middle-income countries; Processing delays; and Limited certification of causes of deaths	System-wide interventions to improve completeness and timeliness of civil registration and vital statistics; New methods for determining causes of deaths including verbal autopsies; and Active surveillance of vital events to generate prospective mortality data in: - Small population in a selective geographic area (Demographic Surveillance System) - Representative set of randomly selected population clusters (Sample Vital Registration System)
**Surveys**					
Deaths in specific age groups	Household visits, asking respondents to list deaths having occurred among some of their relatives (such as siblings)	Every few years; annual in some countries (for example in Senegal)	Under-five mortality rates; Probabilities of adult death: _35_q_15_ or _45_q_15_; Pregnancy-related mortality ratio; and Proportion of adult deaths due to accidents	Limited statistical power only yields average estimates for long reference period before survey; Non-sampling errors (for example misreported ages and dates, omissions) might bias resulting mortality estimates; Limited data on characteristics and circumstances of deaths preclude disaggregation; and Limited coverage of mortality at older adult ages (older than 60 years)	Administering surveys by mobile phones to increase periodicity; Inquiring about additional relatives and social networks to cover older age groups or increase statistical power; Adding questions to better describe socioeconomic characteristics of deceased and circumstances of deaths; and Adding questions about registration status of reported deaths
**Censuses**					
All deaths across the country	Household visits, asking head of household or other informant to list deaths among household members in past 12 months	Approximately every 10 years	Crude death rate; Age-and-sex- specific rates across the life course; and Pregnancy-related mortality ratio	Non-sampling errors (e.g. misreported ages and dates, omissions) might bias resulting mortality estimates; and Limited data on characteristics and circumstances of deaths preclude disaggregation	Extending recall period beyond past 12 months; Including verbal autopsies in post-enumeration surveys to investigate causes of adult deaths; Adding questions to better describe socioeconomic characteristics of deceased and circumstances of deaths; and Adding questions about registration status of reported deaths

Note: _35_q_15_ is the conditional probability of dying between ages 15 and 49 years; _45_q_15_ is the conditional probability of dying between ages 15 and 59 years.

Efforts to strengthen civil registration and vital statistics systems are under way in many low- and middle-income countries, in part hastened by the adoption of SDG targets 16.9 and 17.19.2(b), on providing legal identity for all, and on ensuring that at least 80% of all deaths are registered in each country. Such initiatives entail, for example, digitalizing registration processes; revising the legal frameworks that codify registration of vital events; training additional physicians and personnel in cause of death certification; establishing proactive registration processes; strengthening capacity to publish and use vital statistics; and promoting links between registration authorities and the health sector.^4^^,^^5^ Nonetheless, civil registration and vital statistics interventions require time and sustained funding to reach targeted levels of registration completeness.

Meanwhile, representative mortality data are also collected in low- and middle-income countries from periodic surveys and censuses (Table 1). These inquiries document adult mortality by asking respondents to list their siblings or household members, to indicate their survival status (alive or not) and to briefly describe recorded deaths (such as age at death, date of death). The resulting data allow for calculating several mortality rates directly, including key indicators of adult mortality (for example the probability of dying between ages 15 and 59 years). Additionally, repeated surveys have been used to evaluate the number of lives saved by major health or social programmes.^6^ These data sources all constitute central input in the complex statistical models that produce periodic updates of global health metrics.

However, the sporadic nature of surveys and censuses make them ill-suited to monitor adult mortality trends in real-time and to detect short-term or localized mortality fluctuations caused by epidemics, conflicts or disasters. Censuses are conducted every 10 years at best, and surveys generally about every five years. The sample size of DHS and similar surveys rarely yields more than a few hundred reports of adult deaths. As a result, the data generated allow analysts to estimate only average adult mortality levels over reference periods spanning the past 7–8 years before the survey. This task is further complicated because survey and census data on adult mortality are affected by pervasive non-sampling errors, including incomplete lists of siblings or household members, or misreported ages and dates.^7^

Moreover, current surveys and censuses only partially cover several important aspects of adult mortality. They sometimes include questions about selected circumstances of adult deaths, such as their timing relative to pregnancy and childbirth, or whether they were due to accidents. However, they do not inquire about other circumstances or about pre-mortem exposures of the deceased that might help proxying the underlying cause(s) of death, such as smoking. The socioeconomic characteristics of deceased adults, such as their residence or educational attainment, are seldom ascertained. This gap precludes disaggregating adult mortality indicators between population groups and/or administrative units. Because current surveys often focus on reproductive ages, they generate very limited data on deaths at older adult ages. For example, although more than 40 000 women were interviewed during the DHS conducted in 2018 in Nigeria, only 56 deaths for people older than 60 years were reported.

The Measuring Adult Mortality Community of Practice seeks to facilitate the adoption of improved measurement strategies in low- and middle-income countries, where available population-level data sources on adult deaths are currently partial and/or imperfect, while maintaining comparability and continuity in monitoring adult mortality. So far, the network has organized several webinars for members and other interested parties, and published briefs on recent innovations in survey and census methods. Those events and publications showcased the potential benefits of adding questions to existing survey and census instruments, or of adopting new modes of data collection (Table 1). For example, asking survey respondents not only about the survival of their siblings, but also about their parents, helps better cover mortality at older adult ages during surveys.^8^ Adding questions about deaths among friends and acquaintances increases the number of deaths reported per survey interview, thus enabling the production of estimates of adult mortality for very recent time periods, and possibly for small areas.^9^ Conducting surveys by mobile phone allows monitoring adult mortality more frequently, at lower costs, and during health crises such as the COVID-19 pandemic.^10^ These methodological improvements have so far primarily been tested in limited research investigations. By connecting researchers with national statistical offices and other stakeholders, the network might help accelerate their diffusion and implementation in large-scale national studies.

Fully functional civil registration and vital statistics systems are needed to meet the information needs of policy-makers in various sectors, such as health and education, in the post-2030 era, and to ensure access to essential rights in low- and middle-income countries. However, there are concerns that investments in interim methods of data collection, such as surveys and censuses, might unintentionally leave vulnerable populations excluded, divert scarce resources away from civil registration and vital statistics systems, reduce the perceived need for improving death registration and stall or even reverse progress towards registration targets. To prevent such a vicious cycle, the Measuring Adult Mortality Community of Practice seeks to develop complementarities between data collection systems. To do so, the network facilitated exchanges about the potential use of verbal autopsies (that is, detailed questionnaires probing the symptoms, risk factors and exposures experienced by the deceased) in multiple settings, including in civil registration offices, to determine causes of deaths. The network also organized a webinar covering low- and middle-income countries’ experiences in including questions about the registration status of deaths reported during surveys or censuses. These data assist in exploring whether registration rates vary between population groups, and in investigating the administrative and socioeconomic factors that might limit death registration.^11^ The network also prompted exchanges about the potential of sample vital registration systems, which continuously and actively monitor demographic events in randomly selected population clusters. These platforms have been established only in a few countries, even though they generate representative data on the dynamics of adult mortality and might help expand the registration of adult deaths.^12^

The Measuring Adult Mortality Community of Practice global network might help countries devise innovative strategies tailored for the data needs of their populations, while also outlining steps towards ensuring that mortality data remain comparable and accessible across countries. In a rapidly changing and challenging funding environment, such leadership is needed to sustain progress towards data that adequately reflect the dynamics of adult mortality and ensure faster achievement of universal death registration in low- and middle-income countries.
